# The Effect of Bi-Sr and Ca-Sr Interactions on the Microstructure and Tensile Properties of Al-Si-Based Alloys

**DOI:** 10.3390/ma9030126

**Published:** 2016-02-25

**Authors:** Agnes M. Samuel, Herbert W. Doty, Salvador Valtierra Gallardo, Fawzy H. Samuel

**Affiliations:** 1 Département des Sciences appliquées, Université du Québec à Chicoutimi, Chicoutimi, QC G7H 2B1, Canada; amsamuel@uqac.ca; 2 General Motors Powertrain Group, Materials Technology, Pontiac, MI 48430, USA; herb.doty@gm.com; 3 Corporativo Nemak, S.A. de C.V., P.O. Box 100, Garza Garcia, N.L. 66221, Mexico; salvador.valtierra@nemak.com

**Keywords:** Al-Si-(Cu) alloys, Ca, Bi, Sr, P, modification, demodification

## Abstract

The effect of bismuth and calcium additions on the microstructural characteristics and the tensile properties of the modified and grain-refined Al-Si based B319 alloys were studied in this paper. Based on the results obtained, it has been concluded that Bi reacts with both Sr and Mg, leading to severe demodification of the eutectic Si at Bi levels of 0.15%–0.6% Bi. Bismuth causes a decrease of the yield and tensile strengths for the as-cast and artificially aged conditions and an increase of yield strength in the solution heat-treated condition. The elongation increases with the Bi in the solution heat-treated condition. Based on this, Bi is found to be an efficient solid-solution strengthening element for these alloys. Thus, solution heat treatment, rather than the artificial aging, may be recommended for alloys containing about 1.0% Bi. Calcium has no significant demodification effect on the Sr-modified Si particles at 100–400 ppm Ca, and has a modifying effect at ~600 ppm Ca. The elongation increases with the Ca level at all conditions (as-cast, solution heat-treated, and artificially aged). A slight increase of the tensile strength in the heat-treated conditions was also observed. The lowest tensile properties either in the as-cast or the heat-treated conditions correspond to the most demodified-Si condition obtained at 408 ppm Ca. Calcium is, therefore, not as detrimental to the tensile properties as Bi.

## 1. Introduction

One of the melt treatment procedures normally applied to Al-Si-(Cu) casting alloys is that of modification where, by the addition of certain elements such as sodium (Na), antimony (Sb) or strontium (Sr), the acicular eutectic silicon usually observed in the non-modified alloy is converted to a “modified” or a fibrous form that is beneficial to the mechanical properties of the alloy, in particular, its ductility [1]. To deeply understand the modification process, other factors controlling modification should also be considered, e.g., the presence of various tramp elements, such as Bi, Ca, P, and any possible interactions with the modifier used.

The effect of such impurities on the Sr-modification process, in terms of any interactions between them and the consequent effect on the product integrity, has been studied [1,2,3]. Much research has also been devoted to study the influence of these tramp elements on the structure and the mechanical properties of hypoeutectic Al-Si alloys [4,5,6,7,8,9,10]. With respect to these elements, the tolerable concentrations of Ca and Bi in Al-Si-(Cu) cast alloys are 0.002%–0.004% Ca (20–40 ppm) and Sr/Bi > 0.45, respectively [11]. Bismuth levels of up to 0.5% may be tolerated in wrought alloys to improve machinability: The presence of Bi is known to increase the machining speed and reduce the need for cutting fluids [12]. At certain concentrations, bismuth neutralizes the modifying effect of Na [5,7,10] and Sr [8,9,11]. The addition of Bi to alloy 356.2 also increases the level of microshrinkage [9,10], whereas Ca refines and spheroidizes the iron intermetallics as well as the eutectic silicon in Al-Si base alloys, resulting in improved mechanical properties [1,2]. Calcium was also found to poison the Sb, Na and Sr modification and gives rise to porosity and micro-segregation [1]. Additions of more than 0.05% Ca (500 ppm) to Al-Si alloys is detrimental to the tensile strength and elongation. Fillability and feedability of Al-Si and Al-Si-(Cu) alloys are notably reduced with the addition of Ca [1,2]. In addition, Ca increases the hydrogen solubility in the aluminum melt at trace concentration levels and is often responsible for casting porosity [1].

Studying the effect of Bi and Ca additions on the structure and properties of Al-Si based cast alloys was the objective of several studies done by our research group and other researchers. It has been reported that the effect of impurities and inclusions on the volume fraction of pores is only observable at high hydrogen contents of the melt, 0.3 mL/100 g or more [2,3]. This behavior was later confirmed by further investigations from our group [12]. With respect to gas porosity, hydrogen control is more important than keeping low levels of trace elements (Bi and Ca) provided that the effect of these elements on fabrication and properties is considered. In continuation, therefore, the present work aimed at evaluating the effect of these elements on the tensile properties of Al-Si-(Cu) type 319 alloys, more specifically B319 alloy, a version containing a higher Mg content (0.4 wt %) used in automotive applications, and giving a high response to heat treatment above that obtained with 319 alloy.

However, information on the effect of these elements (as alloying additions) on the mechanical properties of alloys and how these can affect the manufacturing characteristics such as the alloy machinability is not adequate [13] and hence cannot be used as means to control the properties and improve the manufacturing conditions of alloys. Therefore, it is definitely useful to investigate the effect of Bi and Ca additions on the structure and mechanical properties of the Al-Si-(Cu) alloys to fill part of this gap of knowledge. The present work is devoted to this purpose.

In the present work, the alloy melts were produced with low hydrogen levels (degassed with dry argon for twenty minutes using a rotary impeller), resembling the industrial melts obtained by correct liquid metal treatment, believed to be suitable to separate the effect of Bi and Ca on gas porosity from their effect on the mechanical properties. Additionally, the B319 alloy with its higher Mg content was selected due to its high response to heat treatment above 319 alloy. Thus, the results of this study are meant to determine the limits of Ca and Bi to be tolerated in cast alloys prepared from melts with low hydrogen content.

## 2. Experimental Procedure

The B319 alloys were melted in a 30-kg capacity silicon carbide crucible using an electrical resistance furnace. The melting temperature was kept at 750 °C ± 5 °C. The melt was degassed with argon for 20 min using a rotary impeller degassing system (150 rpm) with a flow rate of 5–10 ft^3^/h. The melts were modified using a Al-10 wt % Sr master alloy to get a level of 150–200 ppm Sr in the alloy, and grain-refined using a Al-5Ti-1B master alloy to obtain a ~0.2% Ti level in the alloy. Additions of Bi and Ca were made using Al-5% Bi and Al-10% Ca master alloys. Chemical compositions (using spectroscopic analysis) of the present alloys are listed in Table 1 and Table 2. A total number of 19 compositions were studied. It should be pointed out that, while the concentrations of Bi and Ca used in the present study are higher than those normally observed in the 319 and other such alloys, these high concentrations were employed for the purpose of assessing their influence on the microstructure and properties of the studied alloys. In addition, compared to trace levels, high concentrations are more controllable and reproducible in laboratory experiments.

The melts were poured into a 450 °C preheated permanent mold to produce tensile test specimens [14]. From each alloy composition, 12 sets of tensile test bars were made: Four sets were tested in the as-cast condition, four in the solution-treated condition, and four in the T6 artificially aged condition.

Blue M electric furnace equipped with a programmable temperature controller (±2 °C) was used for solution and aging heat treatments. The solution heat treatments were carried out for 8 h at 495 °C. The solution heat-treated samples were quenched in warm water (60 °C). Eight tensile test bars forming each alloy composition were solution heat-treated. Four bars from each condition were artificially aged at 155 °C for 5 h (T6 temper). Tensile bars were tested at room temperature using an MTS 810 Servohydraulic mechanical testing machine at a strain rate of 4 × 10^−4^. An extensometer with a 50.8-mm gage length was attached to the test bar to measure percentage elongation as the load was applied. In each case, 5–10 bars were tested to obtain standard deviation close to ±5%–7%.

Samples for metallography and image analysis were taken from the shoulder of the test bars to represent each condition. The prepared samples were used to study the microstructure and to carry out the Si particle characterization, using a Clemex Image analyzer in conjunction with an optical microscope. The Si particle characteristics were investigated for the as-cast and the solution heat-treated samples, with a total number of 38 conditions. Tensile tests were carried out for the as-cast, solution heat-treated and the artificially-aged specimens using the MTS machine. Selected samples were sectioned from the tensile bars (10 mm below the fracture surface). Samples were polished and examined for intermetallics using an Hitachi-SU 8000 FESEM microscope equipped with energy dispersive X-ray spectroscopy (EDS) and wavelength dispersive spectroscopy (WDS) systems.

## 3. Results and Discussion

In this study, only the Si particle characteristics were taken as a measure of the effect of Bi and Ca on the microstructure; no attention was paid to the Fe-intermetallic phases, the Cu-phases or the porosity level. The effect of Bi and Ca on these microstructural features was covered in previous publications [2,3,4,12]. Thus, in the following sections, the effect of Bi and Ca as monitored through the Si particle characteristics will be presented.

### 3.1. Bismuth

**Figure 1:**
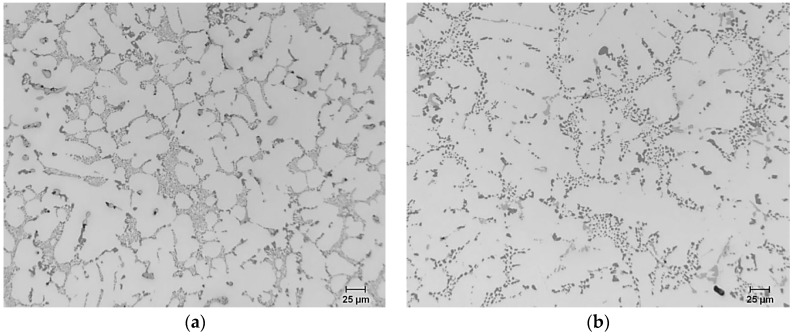
Microstructure of B319 alloy: (**a**) in the as-cast condition; (**b**) after solution heat treament.

Figure 1 shows the microstructure of the base alloy in the as-cast (Figure 1a), and heat-treated conditions (Figure 1b). It is clear that, for the base alloy, the microstructure is fully modified in the as-cast condition. The Si particles have coarsened in the solution heat-treated condition. This is reflected by the variation of the Si particle morphology, where the average particle area increased from 3.66 to 6.67 μm^2^ by solution heat treatment (Table 3).

**Figure 2:**
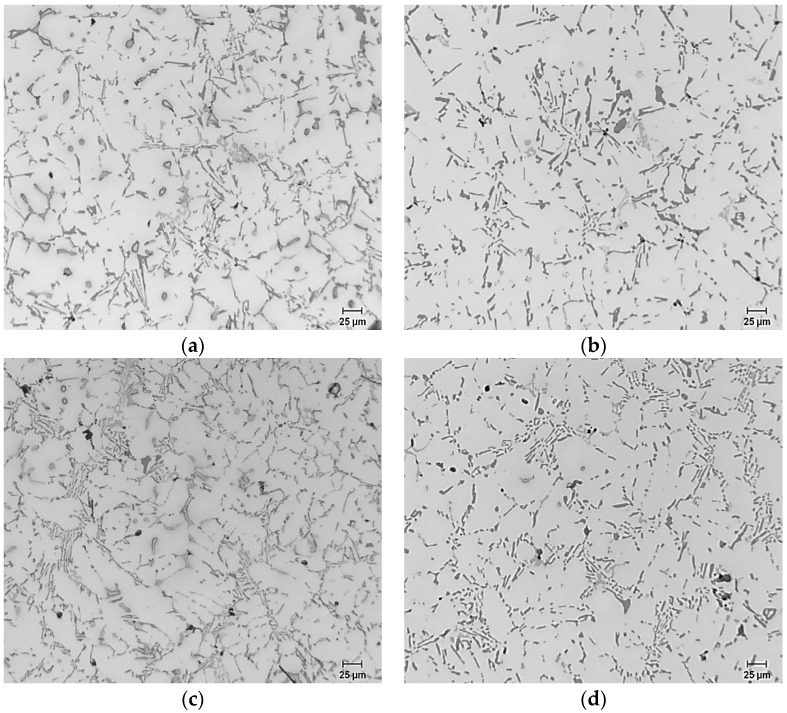
Microstructure of B319 alloy samples containing: (**a**) 0.1% Bi, as-cast; (**b**) 0.1% Bi, solution heat-treated; (**c**) 1%Bi, as-cast; (**d**) 1%Bi, solution heat-treated.

The addition of 0.15%–0.7% Bi to the alloy, counteracted the modifying effect of Sr as shown in Figure 2, where large acicular Si particles dominate in the microstructure. The corresponding data on the average particle area showed a jump of particle areas from 3.66 μm^2^ in the Bi-free alloys to up to 18.1 μm^2^ in the Bi-containing alloys. In addition, particle lengths increased from 2.7 μm in the base alloy to 6–8 μm after Bi addition. Particle roundness and density decreased accordingly (Table 3). The response to heat treatment can hardly be observed (if any) for the lower levels of Bi and started to become more evident at 0.61 % and 0.7% Bi. This can be seen in the data of Table 3.

**Figure 3:**
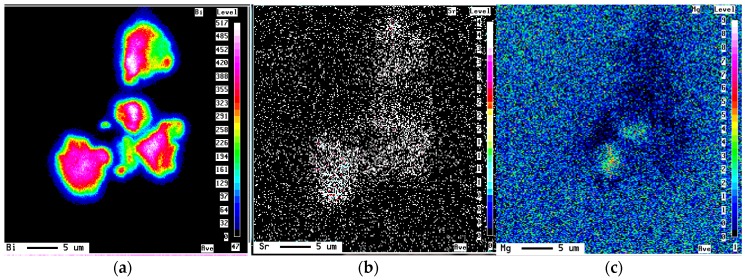
Bi-Sr-Mg interaction in B319 alloy containing 1%Bi: (**a**) Bi; (**b**) Sr; (**c**) Mg distribution.

At higher levels of Bi, *i.e.*, 0.96% to >1.0% (Figure 2), long Si particles were observed in the as-cast condition, as can be seen from Figure 2a. A better response to solution heat treatment was observed (Figure 2d) corresponding to 0.1% Bi. Although finer particles were observed in the as-cast condition, they were acicular in morphology, as can be inferred from the particle aspect ratio and roundness, 2.6 and 0.36, respectively. This phenomenon of demodifying (fading of modification) the Si particles at lower level of Bi in Sr-modified alloys and modifying them at higher levels has been previously observed. However, the level at which the Bi started to operate as a modifier is dependent on the Mg content in that it increases with the increase of Mg content. This took place at about 0.96% to >1.0% Bi in the current alloys (Figure 2c,d), containing about 0.6% Mg and 150–200 ppm Sr. Figure 3 exemplifies the Bi-Sr-Mg interaction in the B319 alloy containing approximately 1% Bi.

**Figure 4:**
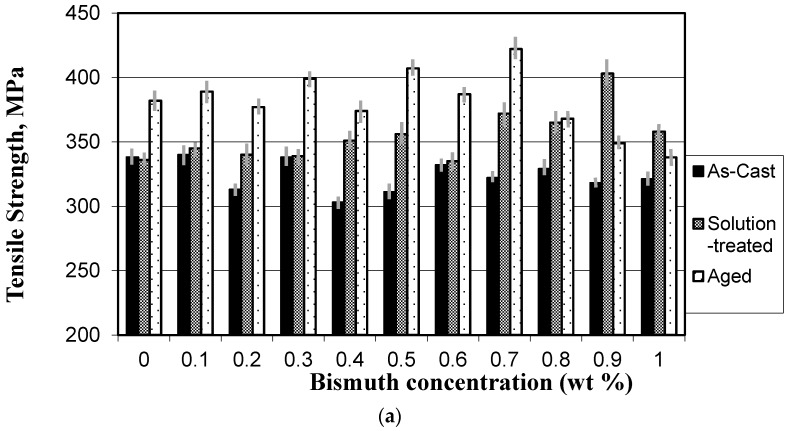

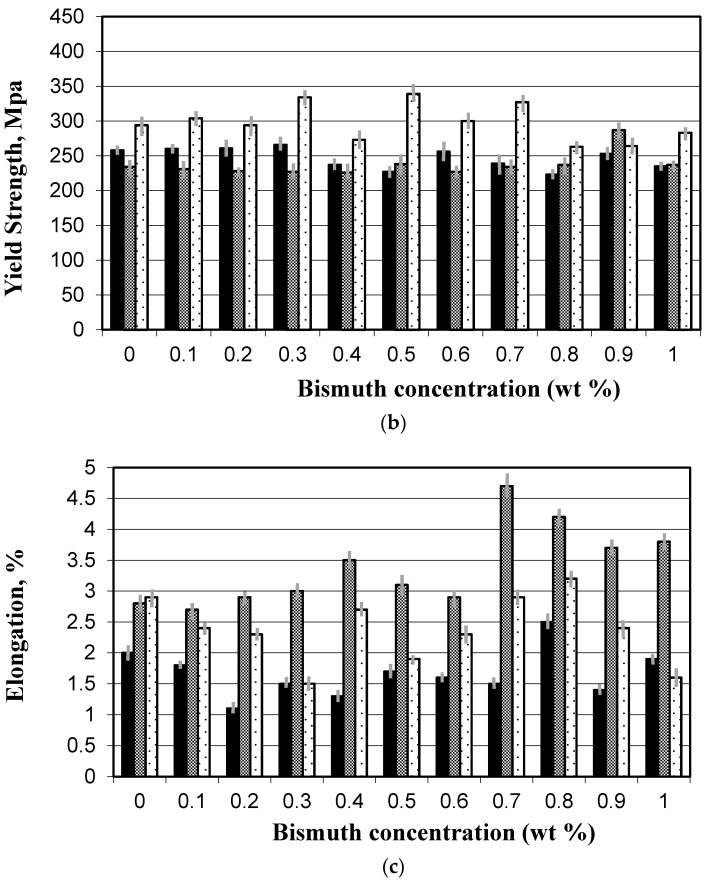
Tensile propties of 319 alloy containing Bi: (**a**) Tensile strength; (**b**) Yield strength; (**c**) Percent elongation.

The tensile properties of the Bi-containing alloys are shown in Figure 4. The ultimate tensile strength (UTS) decreases with the increase of Bi level in the as-cast condition, and this may be attributed to the demodification effect of Bi on the eutectic Si particles, as shown in Table 3, while it increases gradually with Bi level only in the solution heat-treated condition (Figure 4a) due to the fragmentation and spheriodization processes. The observed decrease in alloy strength when Bi concentration is higher than 0.7% may be interpreted in terms of the increase of the volume fraction of Bi-Mg compounds [2,3,4,12] and hence the decrease in Mg content that would contribute to the alloy strength in the form of Mg_2_Si precipitation. On the other hand, the yield strength values of the solution heat-treated specimens are slightly inferior to those of the as-cast specimens for almost all the Bi levels investigated, with the highest values obtained in the artificially aged condition. The yield strength (*YS*) levels of the artificially aged samples decrease with the increase in Bi beyond 0.8% level, while those of the solution heat-treated condition are seen to be independent of the Bi content. In general, the yield strength of the artificially aged samples is higher than those of the as-cast or solution heat-treated samples (Figure 4b). It is clear from Figure 4c that the ductility (%El) in the solution heat-treated condition is higher than those of as-cast and artificially aged conditions at almost all the Bi levels investigated. The elongation of the solution heat-treated condition increases as the Bi content increases up to 0.7%, while relatively no appreciable changes in the elongation are seen to take place in the as-cast or the artificially aged conditions. Thus the reason for this behavior is expected to be due to the formation of Bi-intermetallic compounds during solidification [3,9] and during the aging treatment (see Figure 3), which consume both the Bi and Mg available in the matrix, leading to degradation in their strengthening potential, and the latter is restored after the solution heat treatment.

Previous investigations [2,3,4,15] have shown that the Bi-Mg-Sr interaction, which consumes the amount of Mg required to form the Mg-hardening phases and Sr for Si particle modification, is responsible for the reduction caused in the strength properties of Bi-containing alloys in the heat-treated conditions. The level at which the Bi started to operate as a modifier is dependent on the Mg content in that it increases with the increase in Mg content. In the present alloys with about 0.6% Mg and 150–200 ppm Sr, this takes place at about 0.96% to >1.0% Bi; below this level, Bi acts as a demodifier. This is accompanied by a decrease in the yield and tensile strengths for the as-cast and artificially aged conditions and an increase in yield strength in the solution heat-treated condition. In addition, the elongation increases with the Bi only in the solution heat-treated condition. The interesting aspect of this is the simultaneous increase in ductility (elongation) and strength at the same time (Figure 4).

The observed gradual increase in tensile properties of the solution heat-treated specimens, which cannot be attributed to any of the Si particle characteristics of Table 3, suggests that the Bi is an efficient solid-solution strengthening element for the B319 alloys. The reason for this behavior is expected to be the formation of Bi-intermetallic compounds during solidification [3,9] and during the aging treatment, which consume the Bi available in the matrix and thus degrade its strengthening potential; the latter is improved after solution heat treatment. Based on this, the solution heat treatment, rather than the artificial aging, may be recommended for alloys containing about 1.0% Bi, since the former produces a much higher elongation and strength levels comparable with those of the age-hardened condition.

### 3.2. Calcium

**Figure 5:**
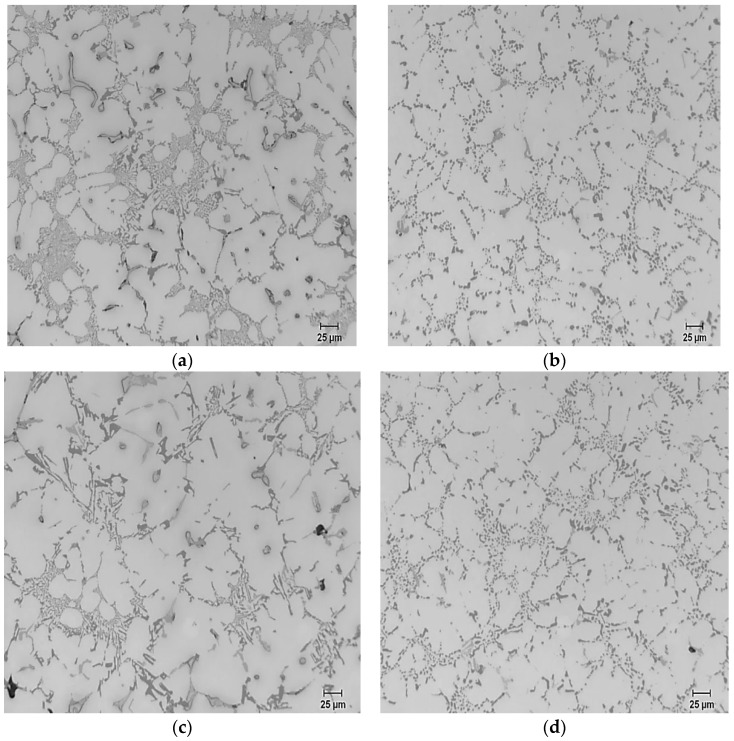
Microstructure of B319 alloy containing: (**a**) 50 ppm Ca, as-cast; (**b**) 50 ppm Ca, solution heat-treated; (**c**) 500 ppm Ca, as-cast; (**d**) 500 ppm Ca, solution heat-treated.

Figure 5 shows the effect of Ca level and solution heat treatment on the microstructure of the B319 alloys. It is clear that up to 51 ppm Ca, no significant variation of the Si morphology is observed either in the as-cast or solution heat-treated specimens compared to the microstructure of the base alloy shown in Figure 1 and data presented in Table 3. At a higher level of Ca (97 ppm), tendency for partial demodification started to appear in the as-cast microstructure, which is apparent from the Si morphology data (Table 3). The average particle area and length have increased from 3.2 μm^2^ and 2.61 μm to 4.99 μm^2^ and 3.33 μm, respectively, and as expected, the particle density decreased, accordingly. The responses to the solution treatment were, however, identical, as can be seen from the micrographs of Figure 5b and the Si particle data of Table 3.

Further additions of Ca resulted in more and more partially modified regions of the microstructure, characterized by larger particle sizes and lower particles densities. The largest and least round Si particles were obtained at 400–500 ppm Ca (Figure 5c), where the average particle area is more than 4 times that of the alloy with 51 ppm Ca (13.4 μm^2^ compared with 3.2 μm^2^). The particle density was also very low compared with the other conditions (6550 particles/mm^2^).

**Figure 6:**
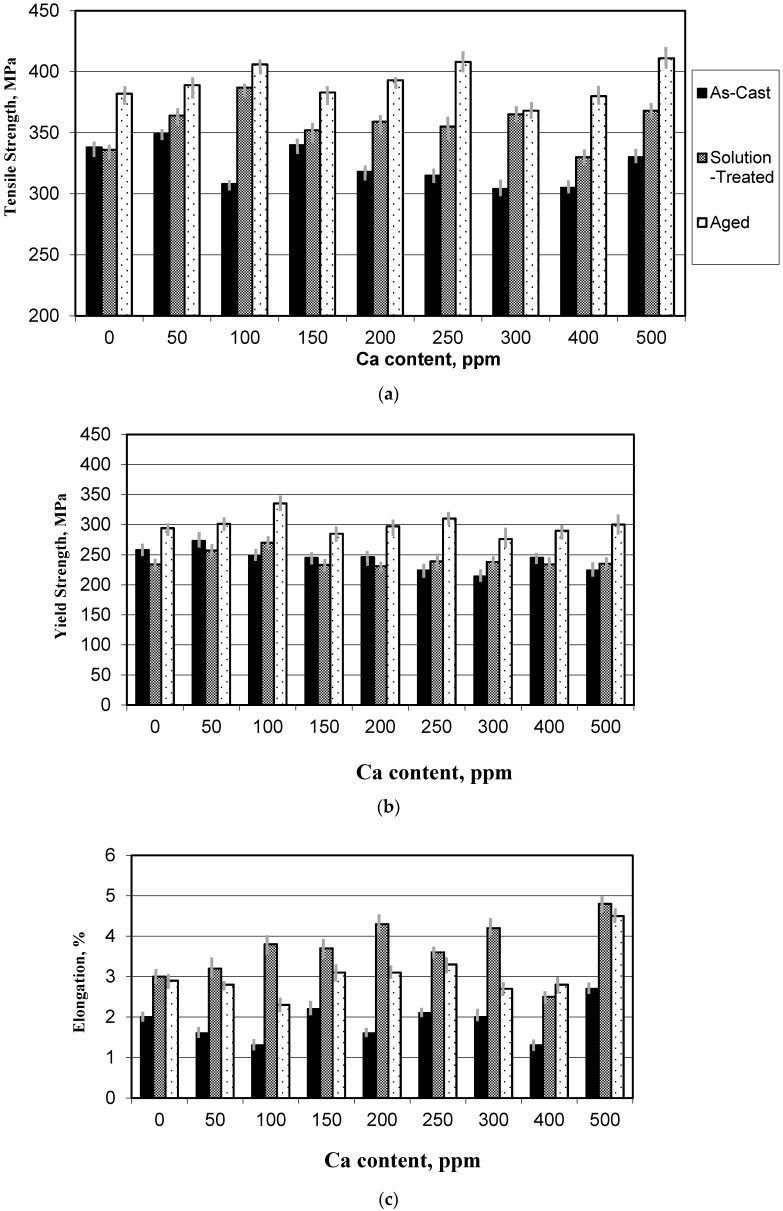
Tensile properties of B319 alloy containing Ca: (**a**) Tensile strength; (**b**) Yield strength; (**c**) Percent elongation.

The effect of Ca level and heat treatment on the tensile properties of the B319 alloys are shown in Figure 6. In the as-cast condition, ultimate tensile strength, the yield strength, and elongation were the lowest values among the conditions studied, especially when the Ca content exceeded 10 ppm due to partial Ca-Sr interaction (Figure 6a). The tensile strength of both solution heat-treated as well as aged conditions slightly increased with Ca level. There is apparently no effect of Ca addition on the alloy yield strength in the solution-treated or aged condition as can be seen from Figure 6b. The effect of Ca level on the performance of the B319 alloys is basically seen in the increase of the elongation at all conditions of as-cast, solution heat-treated and artificially aged conditions (Figure 6c). Of the lowest values of tensile properties at almost all conditions are those obtained at 408 ppm Ca (see Table 1). This Ca level corresponds to the most demodified Si condition in all the Ca-containing alloys (Figure 6). These observations clearly indicate that the effect of Ca on the properties of the B319 alloys is in part due to its demodifying effect on the Si particles caused by formation of Al-Si-Ca-Sr compounds [15].

**Figure 7:**
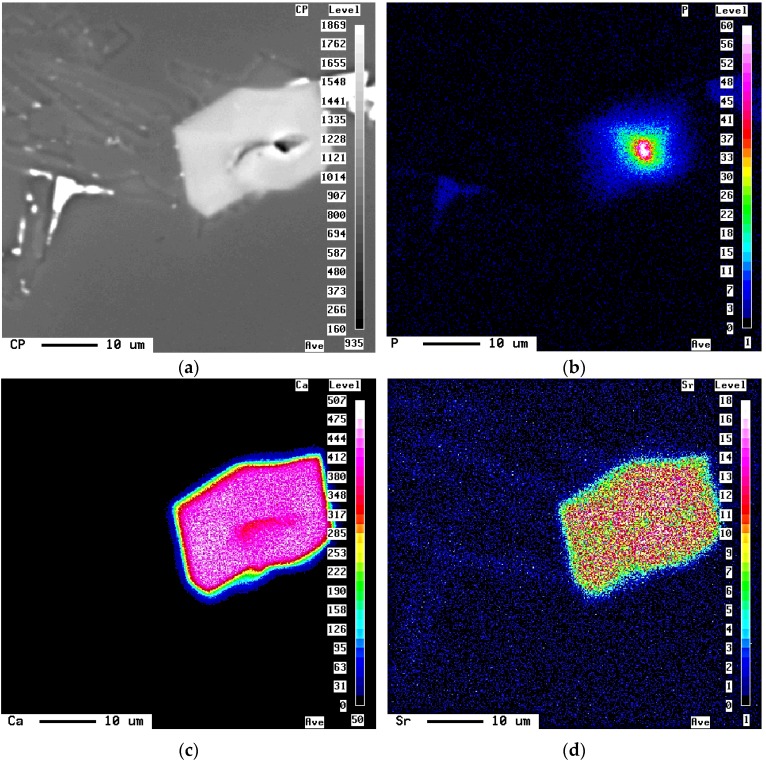
(**a**) Backscattered electron image of B319 alloy sample containing 500 ppm Ca, showing presence of a large Al*x*(Ca,Sr)Si*y* particle and X-ray images showing distribution of (**b**) P; (**c**) Ca; (**d**) Sr in the same.

The role of bismuth (50 to 9000 ppm) and calcium (50 to 200 ppm) additions on the microstructural characteristics in Sr-modified 319 alloys (with/without 0.4 wt % Mg addition) were investigated using optical and electron microscopy and image analysis by El-Haddad *et al.* [12]. The authors found that the modification effect of Sr continuously diminished with Bi addition by up to ~3000 ppm Bi; further Bi addition led to the modification of the Si particles due to the presence of Bi. In the Ca-containing alloys, a coarse eutectic Si structure resulted in Ca additions of 50 ppm, due to the formation of Al*x*(Ca,Sr)Si*y* compounds. Increased Ca additions (up to 200 ppm) did not alter the Si particle size. The Al*x*(Ca,Sr)Si*y* phase particles appeared in rod-like form in the Sr-modified alloys and in plate-like form in the B319 alloys, as shown in Figure 6. MgO, Al_2_O_3_, and AlP particles appear to act as nucleants for the precipitation of the plate-like Al*x*(Ca,Sr)Si*y* phase, see Figure 6. With respect to Ca, its demodifying effect on the Sr-modified alloy was observed above 100 ppm Ca and up to 400 ppm, and its modifying effect at higher concentration, ~600 ppm Ca. The elongation increases with the Ca level at all specimens in as-cast, solution heat-treated and artificially aged conditions. A slight increase in the tensile strength in the heat-treated conditions was also observed. The lowest tensile properties either in the as-cast or heat-treated conditions correspond to the most demodified-Si condition at 400 ppm Ca. The addition of Ca did not lead to an evident decrease of the tensile properties compared to those obtained with the Bi addition (Figure 7), meaning that Ca is not detrimental to the tensile properties as the Bi.

**Figure 8:**
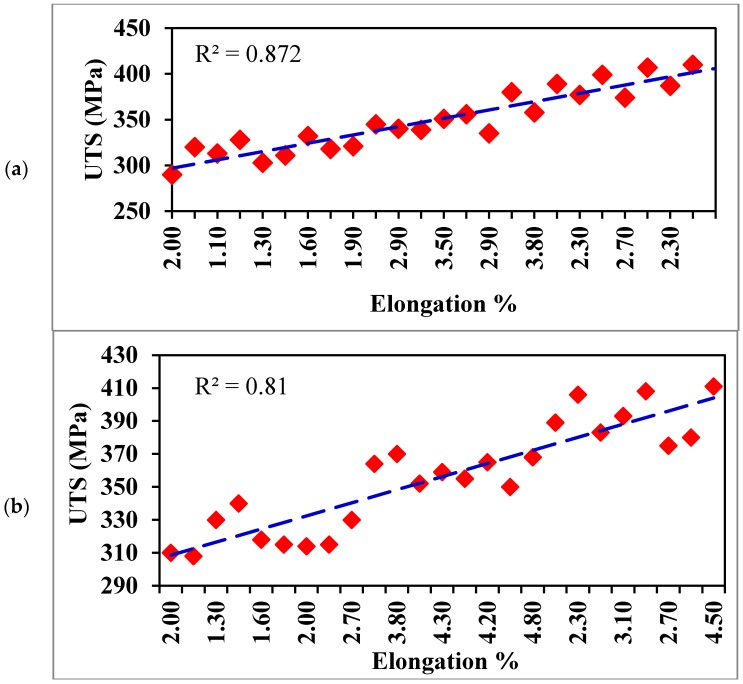
UTS-% El relationships for (**a**) Bi-containing; (**b**) Ca-containing B319 alloys.

Figure 8 shows general UTS-%El relationships for the two series of alloys. It is evident that the addition of Bi up to 1% and Ca up to 500ppm reveals a linear relationship with a high fitting coefficient (>0.8). Applying the concept of quality index [16].

Q = UTS + 150 × logEl
(1)

At maximum attainable elongation (at UTS = 410 MPa), Bi containing alloy shows Q = 465 MPa, whereas Ca alloy exhibits Q = 508 MPa, with a difference of 43 MPa caused by the demodification effect of Bi.

## 4. Conclusions

The effect of Bi and Ca additions and heat treatment on the microstructural characteristics and the tensile properties of the modified and grain-refined B319 alloys were studied for this paper. Based on the results obtained, the following can be concluded:

1. Bismuth acts as a partial modifier for the Si particles at about 0.96% to >1.0% Bi; below this level, it has a demodifying effect.
2. Bismuth causes a decrease in the yield and tensile strengths for the as-cast and artificially aged conditions, and an increase in yield strength in the solution heat-treated condition. The elongation increases with the Bi only in the solution heat-treated condition.
3. The solution heat treatment rather than artificial aging may be recommended for alloys containing about 1.0% Bi.
4. Calcium has a significant demodifying effect on the Si particles in the Sr-modified alloy at concentrations higher than 500 ppm Ca, causing a decrease in the alloy strength.
5. The elongation increases with the Ca level for all alloy conditions (as-cast, solution heat-treated and artificially aged). A slight increase in the tensile strength in the heat-treated conditions was also observed.
6. Calcium, however, is not detrimental to the alloy tensile properties as is the case with Bi.
7. UTS-%El relationships for both Bi- and Ca-containing alloys are linear with a high-fitting coefficient (*R*^2^ > 0.8).

## Figures and Tables

**Table 1:** Chemical composition of the base B319 alloy used in this study (wt %).

Alloy	Si	Cu	Fe	Mg	Ti
B319	6.16	3.67	0.1	0.386	0.13
**Alloy**	**Zn**	**Sr**	**Ti**	**Bi**	**Ca**
B319	0.0017	0.018	0.2	0.0083	0.005

**Table 2:** Chemical composition of the B319 alloys used.

Code	Addition	Bi (wt %)	Ca (ppm)
Base	B319	0.0083	50
1	targeted	0.10	-
	achieved	0.15	48
2	targeted	0.20	-
	achieved	0.26	41
3	targeted	0.30	-
	achieved	0.31	10
4	targeted	0.40	-
	achieved	0.48	11
5	targeted	0.50	-
	achieved	0.61	10
6	targeted	0.60	-
	achieved	0.63	12
7	targeted	0.70	-
	achieved	0.70	14
8	targeted	0.80	-
	achieved	0.96	17
9	targeted	0.90	-
	achieved	0.95	14
10	targeted	1.0	-
	achieved	>1.0	39
11	targeted	-	50
	achieved	0.01	51
12	targeted	-	100
	achieved	0.02	97
13	targeted	-	150
	achieved	0.02	121
14	targeted	-	200
	achieved	0.00	200
15	targeted	-	250
	achieved	0.00	250
16	targeted	-	300
	achieved	0.00	295
17	targeted	-	400
	achieved	0.00	408
18	targeted	-	500
	achieved	0.00	593

**Table 3:** Silicon particle characteristics of the alloys studied.

Condition	As-Cast					Solution Heat-Treated				
	*A*, μm^2^	*L*, μm	*R*	*AR*	*D*, mm^−2^	*A*, μm^2^	*L*, μm	*R*	*AR*	*D*, mm^−2^
Base alloy	3.66	2.71	0.51	1.82	19,000	6.76	3.63	0.56	1.69	17,500
0.15% Bi	16.60	8.14	0.35	2.7	5200	19.7	8.30	0.40	2.68	3800
0.26% Bi	18.10	8.35	0.36	2.70	4500	17.0	7.49	0.41	2.57	4800
0.31% Bi	17.1	8.07	0.37	2.70	4900	21.7	8.34	0.41	2.58	3800
0.48% Bi	14.4	8.02	0.34	2.85	5500	10.6	5.66	0.44	2.36	8000
0.61% Bi	17.7	8.15	0.37	2.77	4750	17.5	7.56	0.41	2.64	6200
0.70% Bi	10.20	6.65	0.35	2.77	8500	9.88	5.17	0.48	2.21	8300
0.96% Bi	7.60	5.65	0.36	2.60	11,500	9.07	4.88	0.48	2.12	8900
>1.0% Bi	8.56	5.75	0.37	2.62	10,000	10.7	5.71	0.44	2.37	8300
051 ppm Ca	3.2	2.61	0.59	1.86	28,000	7.91	3.94	0.56	1.70	11,000
097 ppm Ca	4.99	3.33	0.48	1.98	18,000	7.52	3.90	0.56	1.73	11,000
121 ppm Ca	3.70	3.02	0.48	1.96	23,000	7.80	4.12	0.53	1.84	10,000
200 ppm Ca	7.20	4.43	0.43	2.16	11,000	8.55	4.28	0.54	1.82	9100
250 ppm Ca	5.66	3.99	0.44	2.09	15,000	7.38	4.00	0.54	1.80	10,000
295 ppm Ca	4.48	3.45	0.45	2.02	17,000	7.62	4.12	0.53	1.85	10,000
408 ppm Ca	13.4	6.39	0.40	2.34	6550	13.3	5.91	0.47	2.17	7100
593 ppm Ca	6.51	4.33	0.42	2.13	15,000	7.08	4.00	0.53	1.85	11,000

*A*: Mean particle area; *L*: Mean particle length: *R*: Mean particle roundness; *AR*: Mean particle aspect ratio; *D*: Mean areal density.
